# Synthesis of Polyvinyl Alcohol/Coal Fly Ash Hybrid Nano-Fiber Membranes for Adsorption of Heavy Metals in Diesel Fuel

**DOI:** 10.3390/nano13101674

**Published:** 2023-05-18

**Authors:** Jun Cong Ge, Guirong Wu, Guangxian Xu, Jun Hee Song, Nag Jung Choi

**Affiliations:** 1 Division of Mechanical Design Engineering, Jeonbuk National University, 567 Baekje-daero, Deokjin-gu, Jeonju-si 54896, Republic of Korea; jcge@jbnu.ac.kr (J.C.G.); wgr@jbnu.ac.kr (G.W.); 2 Department of Convergence Technology Engineering, Jeonbuk National University, 567 Baekje-daero, Deokjin-gu, Jeonju-si 54896, Republic of Korea; xgx1996319@gmail.com

**Keywords:** coal fly ash, heavy metals, diesel fuel, ultra-fine filtration, nano-fiber

## Abstract

Some studies have shown that the heavy metal emissions (HMEs) emitted from diesel engines can seriously threaten human health. HMEs are mainly related to the content of heavy metal ions in diesel fuel. Therefore, in order to reduce HMEs from diesel engines, a nano-fiber membrane filtration technology for diesel fuel was investigated. Herein, coal fly ash (CFA) from coal-fired power plants combined with polyvinyl alcohol (PVA) was successfully fabricated into nano-fibrous membranes using green electrospinning technology. In order to evaluate the adsorption properties, various hybrid membranes with different mixing ratios (PVA/CFA = 10/0, 10/1, 10/3, 10/5, and 10/7 by weight) were fabricated. The results show that eight metal ions with different concentrations are found in the diesel fuel, including Pb, Cu, Zn, Al, Fe, Cr, Ba, and Ni. All PVA/FA membranes have different adsorption capacities for metal ions, following the order: Cu > Fe > Pb > Al > Zn > Cr > Ba > Ni. In addition, the adsorption capacity of CFA3 (PVA/CFA = 10/3) is the largest. The super lipophilicity of the PVA/FA membranes also provide more adsorption sites for the contact of HMs with the membranes. The above research results provide guidance for development of ultra-fine filters in the future.

## 1. Introduction

With the large consumption of fossil fuels, the harmfulness of micro-particles and heavy metals (HMs) in the air has attracted increasing attention [1]. HMs, in particular, which are persistent in the environment, are difficult to remove once they are discharged into the atmosphere, and directly pollute the air, water, and soil. Long-term exposure to high concentrations of HMs in the environment leads to developmental stunting, cancer, kidney damage, and other diseases in humans [2]. Relevant research shows that the heavy metal emissions (HMEs) emitted from internal combustion engines (ICE) are mainly related to the following: (1) fossil fuel combustion; (2) lubricating oil combustion; (3) friction between engine parts; and (4) exhaust aftertreatment devices [3,4]. Among these factors, the increased combustion of fuel is the main reason for the increase in HMs in exhaust emissions.

Metal ions contained in liquid fuel not only lead to catalyst poisoning in exhaust post-treatment devices and failure, but also worsen combustion and increase soot emissions, mechanical impurities, and mechanical wear [5,6]. It is difficult to avoid contact with metal devices during the production, transportation, and storage of liquid fuels. In addition, the use of some metal-based additives to improve fuel characteristics can lead to the invasion of metal ions [5]. Biodiesel, in particular, requires multiple water washes during the preparation process to remove glycerol and other impurities, and some metal ions are easily mixed in this washing process [7]. In addition, biodiesel exhibits hygroscopicity and corrosiveness, and contact with metals such as copper, zinc, and lead can lead to an increase in the concentration of metal ions in the fuel [8]. To reduce pollution emissions from diesel engines, South Korea implemented a renewable fuel standard (RFS) policy on 31 July 2015, which requires the mandatory addition of 2.5% biodiesel to diesel fuel, and the blending ratio of biodiesel is expected to reach 5% by 2030 [9]. Prado et al. found some divalent metal ions, such as copper, nickel, and zinc, in both diesel and biodiesel using an inductively coupled plasma atomic emission spectroscopy (ICP-AES) analysis method [10]. Pulles et al. [3] used ICP-MS and Q-APEX to measure the metal concentration in different gasoline and diesel fuels from different countries. They reported that the content of Se, Hg, and As in exhaust emissions is mainly related to fuel combustion, while Zn, Cu, Cd, Pb, Cr, and Ni are mainly related to the combustion of lubricating oil. In addition, the amount of lubricating oil involved in combustion is very small for vehicles without any faults, so the HMs emitted by burning fuel is quite large compared to burning lubricating oil, i.e., about 750 times more. Wang et al. [11] also pointed out that the content of HMs in diesel fuel has a linear relationship with the content of HMs in exhaust emissions, and the HMs emitted by diesel engines are far higher than those from other industries such as coal-fired power plants. Hong et al. [12] analyzed the HMs contained in road sediments and found that the presence of Cr and Ni is mainly related to the exhaust emissions of gasoline vehicles, while Zn, Cr, Cu, and Ni are mainly related to the exhaust emissions of diesel vehicles. Moreover, the HMs from diesel vehicles are higher than those from gasoline vehicles, because diesel engines are mainly used in heavy transportation vehicles, and their pollution emissions are much higher than those from light vehicles. Vehicle exhaust emissions are the main reason for the increase in HMs on urban roads [13]. Many other researchers have found that there are many kinds of hazardous HMs in diesel engine exhaust emissions [14,15]. Therefore, in order to reduce the content of HMs in diesel engine exhaust emissions, it is necessary to reduce the concentration of HMs in diesel fuel.

Generally, the methods for removing HMs mainly include physical methods (e.g., adsorption, ion exchange, and membrane separation), chemical methods (e.g., electrolysis and chemical precipitation), and biological methods (e.g., bioflocculation and phytoremediation). Among them, adsorption is the simplest and most effective method because of its simple operation, good reversibility, and low cost [16]. The adsorption efficiency and adsorption performance mainly depend on the physico-chemical properties of the adsorbent. The most commonly used adsorbents mainly include activated carbon [17], natural zeolite [18], coal fly ash [19], graphene [20], etc. Among them, coal fly ash (CFA) is a solid waste from coal-fired power plants, which is porous, has excellent ion exchange properties, and is often used as a cheap adsorbent to remove HMs in sewage [21]. However, the direct use of CFA as an adsorbent may cause secondary pollution due to its powdery nature and difficulty in recycling. With the rapid development of nano-technology, membrane filtration technology has also been continuously improved. CFA as a cheap adsorbent is expected to be synthesized into nano-fiber membranes for the filtration industry.

On the other hand, electrospinning is one of the simplest nano-technologies for preparing nano-fiber filter membranes. The electrospun membranes are often used as highly effective adsorption materials based on their advantages, such as high porosity, multi-level pore structure, and large specific surface area [22,23,24]. In addition, the surface of electrospun fibers can also be modified by a variety of functional groups (e.g., amino, carboxyl, hydroxyl) that are conducive to adsorption [16,25]. Hydrogen bonding, electrostatic interactions, hydrophilicity/hydrophobicity, π-π dispersion interactions, etc. directly affect the adsorption ability of adsorbent materials [26]. Yang et al. [27] synthesized chitosan (CS) composite fiber membranes using electrospinning technology, and then studied the removal effects of the CS composite membranes on Cr (VI), Cu (II), and Co (II) in aqueous solution. The experimental results show that the CS composite membrane with amino groups exhibits the strongest adsorption capacity for Cr (VI). Xu et al., synthesized SiO_2_-MgO hybrid membranes using one-step electrospinning technology, and studied the adsorption effect of the membranes on Pb (II), Cu (II), Cd (II), Ni (II), and Zn (II) in wastewater. The results show that the adsorption of hybrid membranes on HMs mainly depends on the surface complexation of hydroxyl groups and ion exchange [28]. Tian et al. [29] first used electrospinning technology to fabricate polyvinyl alcohol (PVA) fiber membranes, and then used glutaraldehyde solution to steam cross-link the PVA fibers. Finally, the adsorption ability of the PVA membranes for copper and lead ions in aqueous solution was studied. The research results show that the electrostatic attraction (physical adsorption and van der Waals forces effect) and hydroxyl groups in the molecular chain (chemical adsorption) play a decisive role in the adsorption of HMs.

Many electrospinning membranes have demonstrated excellent adsorption capabilities in removing HMs from aqueous solutions based on their physical and chemical adsorption capabilities. However, the research on electrospun nano-fiber membranes for the removal of HMs from oil or fuel is still lacking. Therefore, in the current work, coal fly ash (CFA) as an adsorbent and polyvinyl alcohol (PVA) as a polymer carrier were successfully electrospun into environmentally friendly composite fiber membranes with different weight mixing ratios. The adsorption capacity of different membranes for heavy metals in commercial diesel fuel was compared and analyzed. Based on the physical and chemical properties of the composite fiber membranes, the adsorption mechanisms of the membrane for heavy metals in oil, including physical adsorption and chemical adsorption, were clarified. This study provides important reference value for further studies on the impact of diesel fuel before and after purification on heavy metals in diesel engine exhaust emissions.

## 2. Experimental Details

### 2.1. Experimental Materials

Polyvinyl alcohol (PVA) 2000 was used as the polymer carrier without any reprocessing, which was purchased from Daejung Chemicals, Co., Ltd., South Korea. Coal fly ash (CFA) as a porous adsorption additive was purchased from Korean K Company. Pure water was used as the solvent. The diesel fuel was purchased from SK OIL petrol station in the Republic of Korea. No other harmful chemical reagents were used during electrospinning.

### 2.2. Preparation of PVA/CFA Hybrid Nano-Fiber Membranes

**Figure 1:**
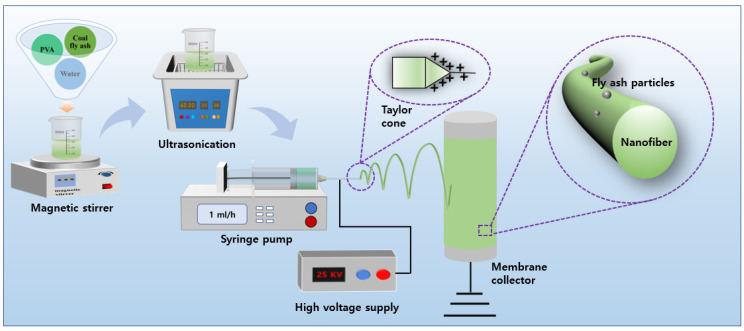
Electrospinning system.

An electrospinning process was utilized to prepare the PVA/CFA hybrid nano-fibers. To remove impurities from the CFA, the CFA was first washed 3 times with deionized water and dried at 105 °C for 2 h. To avoid clogging the nozzle with CFA as a result of the larger particle size during the spinning process, the CFA was ground using a ball mill for 12 h. In total, 2 g of PVA was added to 18 g of pure water to prepare a 10% PVA solution, which was magnetically stirred at 80 °C for 4 h. After all the PVA polymers were dissolved in pure water, a certain amount of CFA was added to the PVA solution and then magnetically stirred for 12 h. The weight ratios of PVA to CFA were 10:0, 10:1, 10:3, 10:5, and 10:7, which were coded as CFA0, CFA1, CFA3, CFA5, and CFA7, respectively. The main spinning parameters, including spinning voltage, spinning distance, and feed speed, were set to 25 kV, 150 mm, and 1 mL/h, respectively. A 12 mL syringe with a 21G nozzle were used to transfer the spinning solution. Figure 1 shows a specific schematic diagram of the electrospinning system.

### 2.3. Characterization Method

Field emission scanning electron microscopy (FE-SEM) was employed to observe the pore structure of FA and the surface morphology of PVA fibers and PVA/FA composite fibers. X-ray diffraction (XRD) patterns of the FA and electrospun nano-fibers were carried out on a high-performance diffractometer with the use of Cu Kα irradiation (λ = 0.154 nm) from 10 to 80° (2θ) at a scan rate of 2 min^−1^. Fourier-transform infrared spectroscopy (FT-IR) was used to analyze the chemical composition and crystallinity of all samples with a range of 400–4000 cm^−1^. The fiber diameter was measured using the ImageJ software (ImageJ 1.48v, Java 1.6.0_20, Wayen Rasband, US National Institutes of Health, Bethesda, MD, USA), and an average of 100 random fibers was obtained. The surface area and pore structure of all electrospun membranes were measured using a surface area and pore size analyzer (BELSORP-max, MicrotracBEL Corp., Osaka, Japan).

### 2.4. Mechanical Evaluation

The mechanical properties of all electrospun membranes were investigated using a universal testing machine (AGS-500G; Shimadzu Co., Kyoto, Japan) based on a standard of ASTM D882-12. The load and speed for testing were uniformly set to 250 N and 21 mm/min, respectively. All electrospun membranes with a thickness of 0.165 mm were cut into a “dog-bone” (dumbbell) shape of 20 × 5 mm. The tensile strength (MPa) was calculated by the maximum force (N) at rupture of membranes by the cross-sectional area (m^2^).

### 2.5. Adsorption Experiment

In order to study the content of heavy metals (HMs) in current commercial diesel fuel and the adsorption performance of the prepared nano-fiber membranes, diesel fuel purchased from a local gas station was directly used for experiments without any treatment. In order to facilitate the analysis, all electrospun membranes were cut to 0.01 g, and the diesel fuel used for adsorption experiment was 30 mL. The adsorption experiment was carried out in a C-SKI thermostatic shaker incubator with a stirring speed of 200 rpm for 24 h. The adsorption performance (*Q*) of all electrospun membranes was calculated using the following equation:

$$Q=\frac{\left(C_{o}-C_{a}\right)V}{M}$$

where *C_o_* and *C_a_* are the initial and final metal concentrations (mg/L), respectively; *V* denotes the working volume (L); *M* indicates the mass of electrospun membranes placed into the working solution (g).

Specific test details regarding the content of HMs in the diesel fuel are as follows: 0.02 g oil samples with 10 mL of HNO_3_ were placed into a Teflon vessel and maintained at 25 °C for 12 h. The following day, all incubation samples were digested in a closed vessel microwave system. The detailed digestion method is as follows: (i) The temperature was increased from room temperature to 180 °C in 30 min, which was held for 20 min, before cooling to room temperature once more; (ii) in order to complete the digestion cycle, 1 mL of H_2_O_2_ was added and the mixed solutions were microwave treated (microwave conditions were the same as above); (iii) after the reaction was completed, the mixed solutions were diluted to 50 mL with distilled water and used for the analysis of the elements selected by inductively coupled plasma-optical emission spectrometry (ICP-OES). The different wavelengths of the test metal are shown in Table 1.

### 2.6. Contact Angle Measurement

The wettability and permeability of the as-prepared electrospun nano-fiber membranes on diesel fuel were characterized using an L2004A1 Ossila contact angle analyzer with a measurement accuracy of ±1°. In order to reduce the influence of the surface roughness and gravity effect while improving the measurement accuracy of the contact angle, a 5 µL droplet of deionized diesel oil was deposited on the nano-fiber membrane surface [30]. A high pixel camera (1920 × 1080) was used to capture images of dripping fuel droplets. The diesel fuel contact angle was analyzed using the matching image analysis software (Version 3.0.6.0). During the test, the angles of the left and right sides of the fuel droplets were measured three times each, and then the average value was obtained as the final contact angle.

## 3. Results and Discussion

### 3.1. Characterization

**Figure 2:**
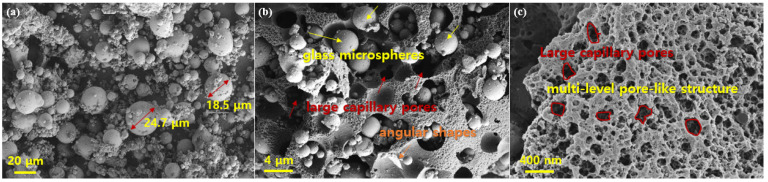
SEM images of CFA before (**a**) and after (**b**) ball milling; the pore structure (**c**).

**Figure 3:**
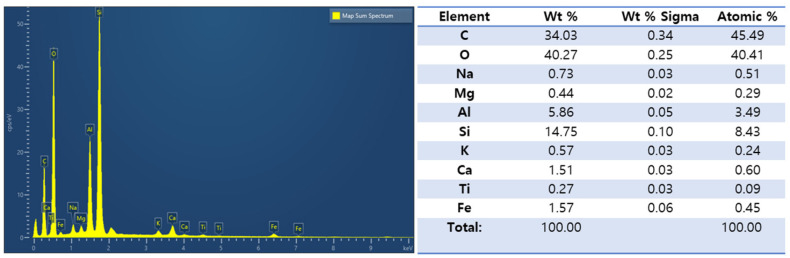
EDS spectra of CFA.

**Figure 4:**
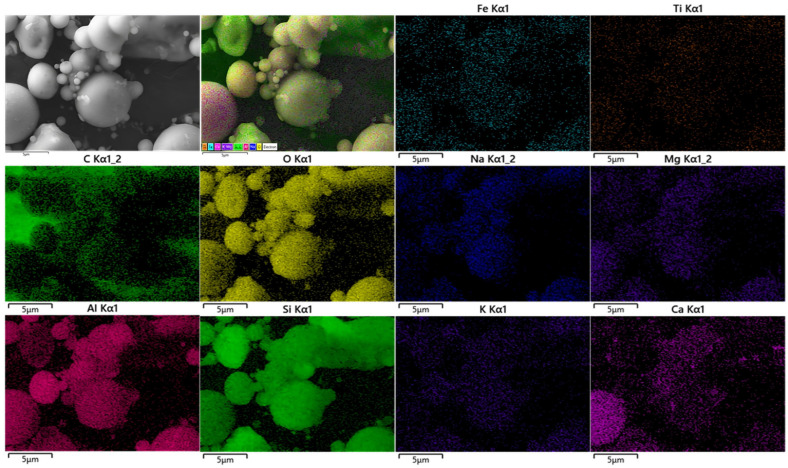
Elemental mapping of CFA.

Figure 2 shows the SEM images of coal fly ash (CFA). As shown in Figure 2a,b, the particle size of fly ash after the ball milling treatment was significantly reduced, even if there were a few large particles, their diameter was still less than 4 μm. The SEM structure clearly shows that CFA was composed of multiple spherical particles and ash, and the surface of the ash had a multi-level pore structure. In addition, as seen in Figure 2c, a multi-level pore-like structure (micropores: <2 nm; meso-pores: 2–50 nm; macro-pores: <50 nm) with large capillary pores (50 nm < d < 10 μm) was also observed on the agglomerated CFA particles, which was mainly related to incomplete oxidation of the precursor coal [31]. Figure 3 and Figure 4 show the chemical analysis of the CFA enabled by EDS spectra and elemental mapping typical results, respectively. According to the results, the CFA was mainly composed of C, O, Al, and Si, in addition to a small amount of Na, Mg, K, Ca, Ti, and Fe. Among the metal elements, the contents of Si and Al were the highest, i.e., 14.75% and 5.86%, respectively. In addition, up to 34.03% unburnt carbon was found in the CFA. The existence of these elements constitutes the main chemical composition of CFA, including SiO_2_, AlO, Fe_2_O_3_, CaO, and a small amount of carbon. The physico-chemical and mineral properties of FA mainly depended on the coal composition, combustion temperature, and collection devices [32]. The chemical composition, such as the unburnt carbon, and the porous structure of CFA are very beneficial to the adsorption of HMs such as Ni, Cr, Pb, As, Cu, Cd, and Hg [33]. In addition, most CFA is alkaline, which causes the surface to have a negative charge at high pH; thus, they have a strong precipitation effect and ion exchange capacity on adsorbing HMs [32]. Therefore, the porous structure and special physico-chemical properties of CFA make it a great prospect as a cheap adsorbent.

**Figure 5:**
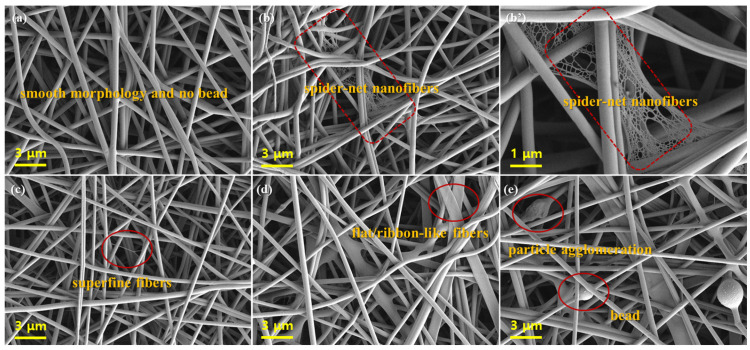
SEM images of (**a**) CFA0, (**b**) CFA1, (**b’**) CFA1, (**c**) CFA3, (**d**) CFA5, and (**e**) CFA7.

Figure 5 presents the SEM images of neat PVA and PVA/CFA hybrid nano-fiber membranes produced by electrospinning. In Figure 5, most nano-fibers had good, smooth, and uniform fibrous structures without beads. The neat PVA nano-fibers were cylindrical, continuous, and randomly distributed, which indicates that the concentration (10 wt%) of PVA in the electrospinning precursor and the spinning parameters were appropriate for the preparation of nano-fibers. Zubair et al., found that 10% PVA was the most suitable spinning concentration for the preparation of homogeneous bead-free nano-fibers. Both too low and too high a spinning precursor results in the appearance of beads and agglomerates on the fibers [34]. As shown in Figure 5b,b’, it can be clearly observed that there were some spider-net nano-fibers between the fibers when 10 wt% CFA was added to the PVA spinning solution. The main components in CFA included SiO_2_, Al_2_O_3_, Fe_2_O_3_, MgO, TiO_2_, etc., which may have been beneficial to promote polymer ionization during electrospinning, resulting in the formation of some super-fine fibers with a diameter of tens of nanometers (see Figure 5c). These results are consistent with [35]. In addition, as shown in Figure 5d,e, continuing to increase the content of CFA in the PVA solution resulted in the formation of some smooth and flat/ribbon-like fibers, and the agglomeration of CFA particles and some beads in the PVA fibers. This may have been because, during the high-voltage electrospinning process (25 kV), the addition of slightly excessive CFA greatly increased the conductivity of the PVA/CFA mixed spinning solution, and finally led to the “over stretching” of PVA fibers during stretching, and thus, the formation of some flat/ribbon-like fibers. In Table 2, the conductivity of the PVA/CFA mixed spinning solution increased from 309.33 to 861.00 μS/cm with the addition of CFA. By comparing the concentration of the spinning solution and spinning parameters, Topuz et al. [36] found that increasing the concentration and conductivity of the spinning polymer and the spinning voltage were beneficial to the formation of flat/ribbon-like fibers.

**Figure 6:**
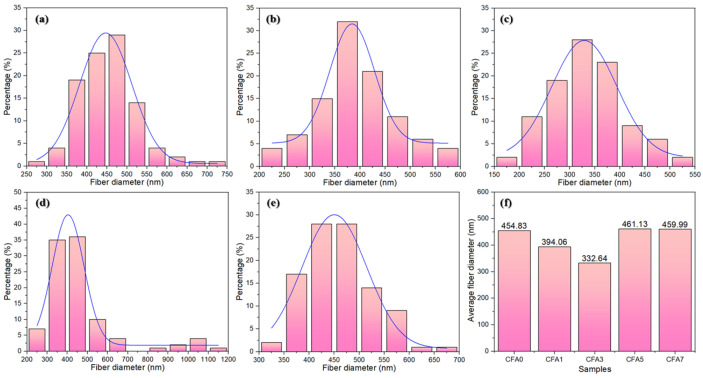
Diameter distributions of (**a**) CFA0, (**b**) CFA1, (**c**) CFA3, (**d**) CFA5, (**e**) CFA7, and (**f**) their average diameter.

**Figure 7:**
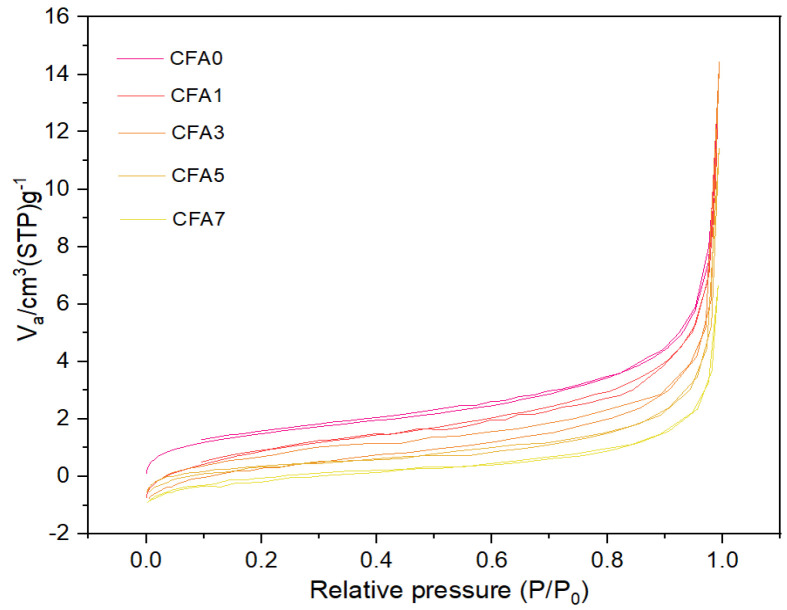
Nitrogen adsorption–desorption isotherms of PVA/CFA hybrid membranes.

Figure 6 illustrates the fiber diameter distributions and average fiber diameter of neat PVA and PVA/CFA hybrid membranes produced by electrospinning. As shown in Figure 6, the average diameter of most fibers was distributed in a narrow range between 300 and 500 nm, which indicates that the fibers prepared by electrospinning were uniform in diameter. In addition, the average fiber diameters of CFA0, CFA1, CFA3, CFA5, and CFA7 nano-fibers were found to be 454.83 ± 76.42, 394.06 ± 77.88, 332.64 ± 73.16, 461.13 ± 187.35, and 459.99 ± 67.23 nm, respectively. As the content of CFA in PVA increased from 0/10 to 7/10, the average diameter of fibers decreased first and then increased, and the average diameter of CFA3 was the smallest. An appropriate increase in the content of CFA resulted in a decrease in the fiber diameter. This may have been related to an increase in the electrical conductivity of the PVA/CFA mixed electrospinning solution and the charge density of the FA particles causing the electrospinning solution to have a stronger extensibility in the electric field and to form thinner nano-fibers. In addition, polymer molecular weight, electrospinning parameters, such as voltage, distance, feed speed, temperature, humidity, and other parameters also affected the diameter of fibers [37]. However, these parameters were constant in this experiment; thus, all the above-mentioned influence factors can be ignored. Figure 7 depicts the nitrogen adsorption–desorption branches of pure PVA and PVA/CFA hybrid membranes. As shown in Figure 7, the isotherms for all membranes were ascribed to Barrett–Joyner–Halenda (BJH) type IV, indicating the typical type for meso-porous structure. Moreover, the relationship between the fiber diameter and specific surface area of PVA/FA composite fibers and the viscosity and conductivity of the spinning solution is shown in Table 2. According to the table, it can be seen that CFA3 had the largest pore volume, pore diameter, and BET, which were 0.022 cm^3^/g, 15.93 nm, and 6.46 m^2^/g, respectively. Fiber membranes with macro-porous properties are more prone to surface functionalization. In addition, the large pore volume and high specific surface area provide more adsorption sites for adsorbing more HMs [38,39].

### 3.2. Chemical Characterizations

**Figure 8:**
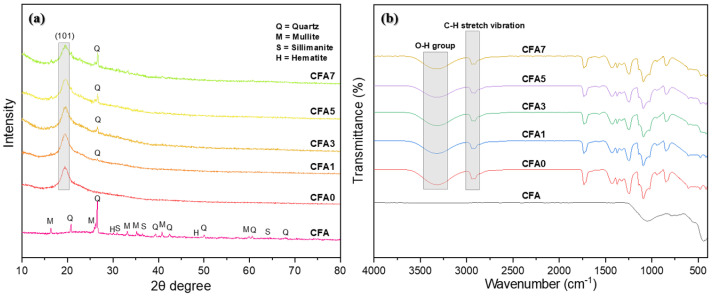
(**a**) XRD and (**b**) FT-IR analysis.

Figure 8a presents the XRD spectra of CFA, the neat PVA membrane, and PVA/CFA hybrid membranes. According to the XRD analysis results, the major crystalline phases in the CFA were quartz (SiO_2_) and mullite (3Al_2_O_3_·2SiO_2_), and a small amount of sillimanite (Al_2_O_3_·SiO_2_) and hematite (Fe_2_O_3_), which is similar to the analysis results from other researchers [40]. For the nano-fiber membranes, a relatively strong and wide diffraction peak was observed around at 20°, which was due to the strong inter-molecular and intra-molecular hydrogen bonding interactions occurring in the (101) plane of the semi crystalline structure of PVA [41]. In addition, quartz crystals were detected in all PVA/CFA hybrid membranes, which confirmed the formation of the nano-composite. Moreover, the peak strength of quartz gradually increased as the amount of CFA added to the PVA solution increased. In particular, when the mixing ratio of CFA and PVA exceeded 3:10, the crystallization of quartz was very obvious. Figure 8b shows the FT-IR spectra of CFA and the PVA/CFA composite fiber membranes. For CFA, there were several typical vibrations that occurred at 1049, 776, and 440 cm^−1^, possibly related to the Si-O-Si asymmetric stretching vibrations of silica, quartz, and silica, respectively [42]. For the nano-fiber membranes, on the whole, the peak positions and intensities of all membranes were almost the same, and there was no obvious difference with the addition of CFA. Moreover, no new peaks were found in the PVA/CFA hybrid membranes with the addition of CFA, which may have been because the peaks of the functional groups of CFA were covered due to the extension of the wider and stronger bond stretches of PVA [43]. However, due to the main component of CFA containing a large amount of silica, the peak strength of the PVA/CFA hybrid membranes at 440 cm^−1^ was slightly weakened with the addition of CFA. Therefore, although there was no obvious physical or chemical interaction between PVA and CFA in the hybrid nano-fiber membranes, the slight change in FT-IR spectra results shows that the hybrid nano-fiber membranes conferred with the blending of PVA and CFA. As shown in Figure 8b, several relatively strong absorption peaks occurred at 3326–3309, 2940–2912, and cm^−1^, which may have been related to the vibrations of O–H stretching from H bonds, the –CH2– and C–H stretching vibration from the methylene and alkyl groups, and C=O carbonyl stretching from the acetate groups, respectively. In addition, the C–H bending vibration of CH2 may have occurred at 1429 cm^−1^; the C–H deformation vibration may have occurred at 1375 cm^−1^; the C–O–C asymmetric stretching may have occurred at 1142 cm^−1^; the stretching vibration of C–O and C–C may have occurred at 1092 and 847 cm^−1^.

### 3.3. Mechanical Properties

**Figure 9:**
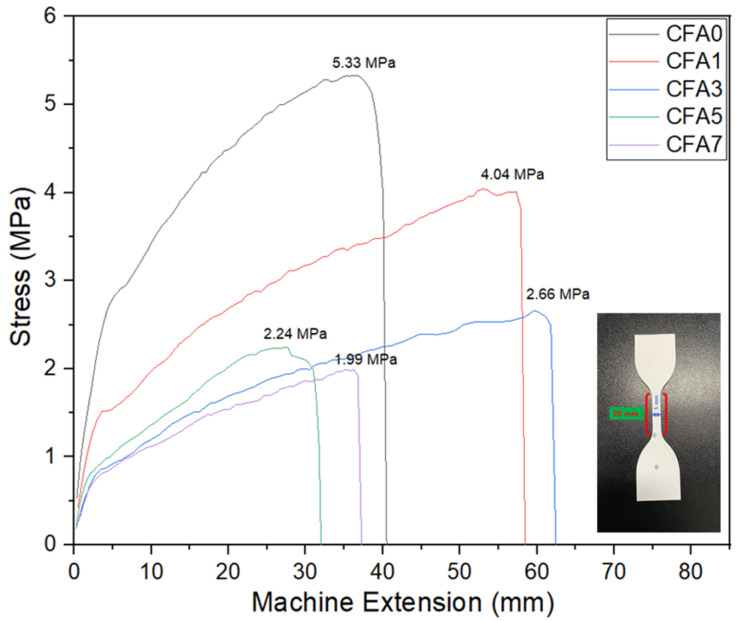
Mechanical properties of PVA/CFA hybrid membranes.

Figure 9 shows the mechanical properties of the PVA/CFA nano-fiber membranes. As shown in the figure, it is clear that the pure PVA membrane had the highest stress of 5.33 MPa, followed by CFA1 at 4.04 MPa, CFA3 at 2.66 MPa, CFA5 at 2.24 MPa, and CFA7 at 1.99 MPa. The PVA membrane shows high toughness during the tensile process (see Video S1 in the Supporting Information). The tensile strength of the PVA/CFA nano-fiber membranes decreased with the increase in the CFA content. This may have been because the addition of CFA reduced the connection between PVA molecules. In addition, when an appropriate amount of CFA (CFA1 and CFA3) was added to the PVA solution, the prepared fiber membranes showed good machine extension. This may have been related to the formation of hydrogen bonds between mixed components or inter-molecular interactions between PVA molecular chains and CFA particles. Although CFA3 did not exhibit the highest tensile strength, its machine extension was the largest compared with the other membranes, and its tensile strength exceeded 2 MPa, which meets the minimum tensile strength required (ca. 2 MPa) for the large-scale processing of membranes. When the mechanical strength is lower than 2 MPa, the membrane is difficult to be further processed and is easily damaged, which limits its practical application [44].

### 3.4. Oil Contact Angle Analysis

**Figure 10:**
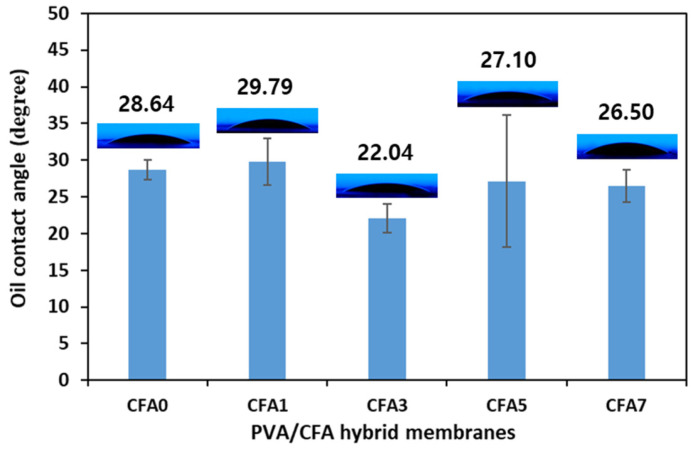
Oil contact angle of PVA/CFA hybrid nano-fiber membranes.

Figure 10 shows the effect of different PVA/CFA hybrid nano-fiber membranes on wettability and permeability by evaluating the oil contact angle (OCA). Generally speaking, the wettability and permeability of a material’s surface are usually evaluated by measuring the contact angle formed between the liquid and the material’s surface. The contact angle is mainly formed when liquid droplets (water or oil) drop from a certain height onto a material in the horizontal direction. The lower the contact angle, the greater the wettability; conversely, the higher the contact angle, the lower the wettability. In general, when the contact angle (Ɵ) is 0°, it indicates perfect wetting; less than 90° indicates good or high wetting; greater than 90° indicates low wetting; equal to 180° indicates non-wetting. In addition, a small contact angle means a high adhesion state due to a large surface area and a high surface energy between liquid and solid surfaces [45]. As shown in Figure 10, the OCAs of CFA0, CFA1, CFA3, CFA5, and CFA7 were 28.64°, 29.79°, 22.04°, 27.10°, and 26.50°, respectively. The OCAs of all the prepared membranes were far less than 90°, which indicates that these nano-fiber membranes had good wetting characteristics. The OCA of CFA3 was the smallest, which indicates that its wettability was the best. The diesel fuel was absorbed very fast, e.g., within a maximum of only 5 s (see Video S2 in the Supplementary Materials). Moreover, with the addition of FA, the wettability of the PVA/CFA hybrid membranes seemed to be improved. The above results may have been related to the large amount of hydroxyl groups (-OH) present on the PVA fibers, the small fiber diameter, the high specific surface area, and the large pore diameter. The low fiber diameter, the large specific surface area, and the large pore diameter resulted in more fuel droplets contacting or passing through the fiber membrane. In addition, CFA contains a large amount of SiO_2_, which is beneficial to change the roughness of the nano-fiber, thereby further improving the wettability of the PVA/CFA hybrid membranes.

### 3.5. Heavy Metals Adsorption Capacity Analysis

The initial concentrations of the eight heavy metals (HMs) in diesel fuel, including Cu, Zn, Pb, Al, Fe, Ba, Cr, and Ni, are shown in Table 3. According to the ICP-OES detection results, the concentration of Cu in these HMs was the highest, i.e., 3.728 ppm, followed by Fe, Zn, Al, Pb, Cr, Ba, and Ni at 3.642, 3.588, 2.712, 2.361, 0.409, 0.256, and 0.109 ppm, respectively. Generally, the existence of these metals is undesirable because they may be harmful to human health and the environment. Many previous researchers pointed out that Pb emitted from diesel engine exhaust emissions have potential carcinogenicity, and Cu, Ba, Cr, Fe, Zn, and Ni will cause gastrointestinal diseases, metal fume fever with symptoms, respiratory issues, and other diseases [46]. In addition, the presence of these HMs may also cause chemical reactions such as oxidation and degradation, thus reducing the quality and shelf life of diesel fuel. These HMs will inevitably be mixed into diesel fuel in the process of oil generation and transportation. Therefore, it is of great significance to find suitable purification methods for improving fuel quality and reducing harmful exhaust emissions from diesel engines.

**Figure 11:**
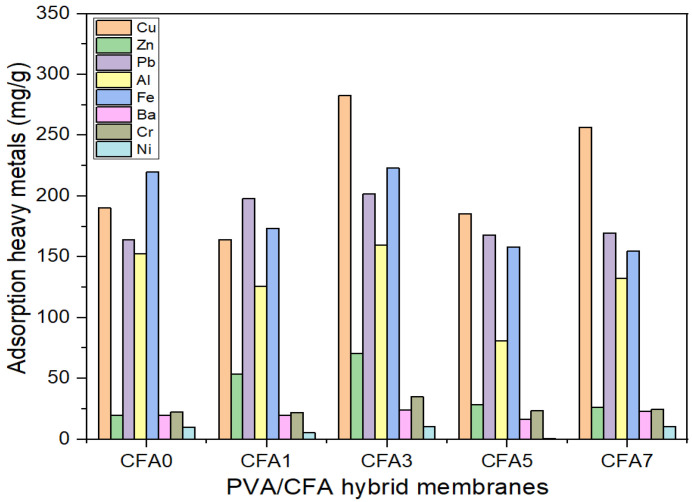
Adsorption capacity of PVA/CFA nano-fiber membranes for heavy metals.

**Figure 12:**
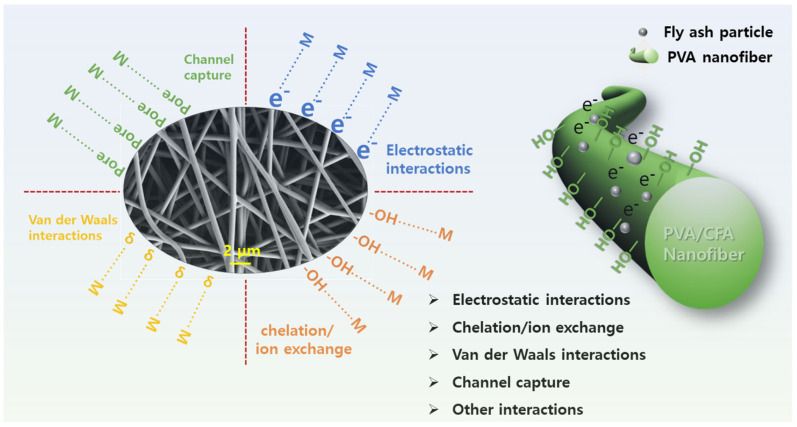
Schematic diagram of adsorption mechanism.

Figure 11 shows the adsorption behavior of the PVA/CFA nano-fiber membranes for the eight HMs mentioned above in diesel fuel. As shown in Figure 11, all the prepared electrospun nano-fiber membranes had different adsorption capacities on the eight HMs. This was even true for the pure PVA membrane, which exhibited high adsorption capacities of 190.38, 163.89, 152.74, and 220.01 mg/g for Cu, Pb, Al, and Fe, respectively. This may have been due to the presence of some hydroxyl groups (–OH) on the PVA fibers, which can serve as a good adsorbent for adsorbing various heavy metals through electrostatic interactions, hydrogen bonding, coordination chelation, cross-linking, and ion exchange [16,29]. In addition, the high BET surface area of the PVA membrane, the many available binding sites, the inter- and intra-pores, and the good wettability and permeability were favorable for the adsorption of heavy metals [8]. Moreover, the good lipophilicity of the PVA nano-fiber membrane was beneficial to the adsorption of HMs, and the large number of hydroxyl functional groups contained in PVA had excellent complexing ability for HMs. On the other hand, the CFA3 membrane showed the highest adsorption capacity for the eight HMs compared with the other fiber membranes. It was able to remove 283.02 mg/g of Cu, 222.96 mg/g of Fe, 201.57 mg/g of Pb, 159.55 mg/g of Al, 70.80 mg/g of Zn, 35.11 mg/g of Cr, 24.27 mg/g of Ba, and 10.46 mg/g of Ni. This may be attributed to the unique physical characteristics of the CFA3 membrane, such as the smallest average fiber diameter (332.64 nm), the largest total pore volume (0.022 cm^3^/g), the largest mean pore diameter (15.93 nm), and the largest BET surface area (6.46 m^2^/g), compared with the other fiber membranes. The smaller the diameter of the fiber, the more numerous and the smaller the pores that formed were. The existence of more small pores causes capillary action, thus improving the driving force of oil molecules entering the fiber [29]. A large BET surface area, large pore size, high pore volume, and multi-stage pore structure provide the opportunity to adsorb HMs [38]. In addition to the physico-chemical properties of PVA nano-fiber membranes, the adsorption capacity of CFA3 was also affected by the characteristics of CFA. Most CFAs are strong alkali materials, and their surfaces are negatively charged. CFA contains a large number of reactive silicate and aluminate groups, resulting in negative zeta potentials on its surface, which strengthens the electrostatic attraction between CFA and positively charged metal ions [47]. Therefore, some HMs can be removed from oil by precipitation or electrostatic adsorption [48]. Moreover, the main components in CFA include Al-Si oxides, Fe-oxides, Ca-oxides, and unburned carbon, which could enhance the adsorption ability of HMs [49]. The adsorption of HMs by all the prepared fiber membranes exhibited the following order: Cu > Fe > Pb > Al > Zn > Cr > Ba > Ni. The reason that the Cu was easier to adsorb may be attributed to the fact that Cu can form complex structures with hydroxyl groups on PVA molecular chains and become hydroxyl groups on nano-fibers through complexation [29]. To sum up, the mechanism for the removal of HMs in diesel fuel is mainly ascribed to physico-chemical adsorption. The physical adsorption is mainly related to the electrostatic interaction between positively charged HMs and negatively charged CFA, Van der Waals interactions, and channel capture from pores. The chemical adsorption is mainly related to chelation and/or ion exchange between HMs and the –OH functional group [29,50]. The detailed adsorption mechanism of PVA/CFA hybrid nano-fiber membranes is shown in Figure 12.

## 4. Conclusions

In this work, polyvinyl alcohol (PVA)/coal fly ash (CFA) hybrid nano-fiber membranes were successfully fabricated via the electrospinning technique for the first time to remove heavy metals (HMs) from diesel fuel. Field emission scanning electron microscopy (FE-SEM) showed that CFA has a porous structure, and the proper addition of CFA is beneficial to reduce the diameter of the PVA fiber and increase the pore volume. Fourier-transform infrared spectroscopy (FT-IR) and X-ray diffractometer (XRD) analysis revealed the structural properties of the PVA/CFA composite. Universal testing machine (UTM) results showed that CFA3 membrane had the highest toughness and a good tensile strength of 2.66 MPa. The contact angle results indicated that all the prepared fibrous membranes had a good wettability to diesel fuel, with CFA3 exhibiting the smallest oil contact angle of 22.04°. In addition, all the prepared fiber membranes had a certain adsorption capacity for the various heavy metals in diesel fuel, and the adsorption order was Cu > Fe > Zn > Al > Pb > Cr > Ba > Ni. Among all the fiber membranes, CFA3 had the highest adsorption capacity, especially for Al, Pb, Fe, and Cu, with an adsorption capacity from 160 to 283 mg/g. To sum up, CFA, an industrial waste, was perfectly combined with environmentally friendly PVA in order to prepare nano-fiber membranes. These exhibited a high adsorption performance and have huge application potential and social benefits in the oil filtration industry in the future.

## Figures and Tables

**Table 1:** Wavelengths used for ICP-OES.

Elements	Wavelengths (nm)
Cu	221.810
Zn	206.200
Pb	220.353
Al	396.152
Fe	261.187
Ba	230.424
Cr	206.157
Ni	221.647

**Table 2:** Comparison of changes in diameters of PVA/CFA hybrid nano-fibers.

Title 1	CFA0	CFA1	CFA3	CFA5	CFA7
Minimum diameter (nm)	280	218	161	266	338
Maximum diameter (nm)	736	592	520	1173	669
Average diameter (nm)	454.83	394.06	332.64	461.13	459.99
Standard deviation (nm)	76.42	77.88	73.16	187.35	67.23
Viscosity (cP)	2130.17	2186.77	2625.33	3603.33	3828.00
Conductivity (μS/cm)	309.33	462.00	624.67	729.67	861.00
Total pore volume (cm^3^/g)	0.018	0.017	0.022	0.014	0.011
Mean pore diameter (nm)	12.87	10.52	15.93	14.86	12.35
BET surface area (m^2^/g)	5.65	5.34	6.46	3.89	3.76

**Table 3:** Initial concentration of heavy metals in diesel fuel.

Heavy Metals	Concentration (ppm)
Cu	3.728
Zn	3.588
Pb	2.361
Al	2.712
Fe	3.642
Ba	0.256
Cr	0.409
Ni	0.109

## Data Availability

Not applicable.

## Supplementary Materials

The following supporting information can be downloaded at: https://www.mdpi.com/article/10.3390/nano13101674/s1, Video S1: Mechanical properties. Video S2: Oil contact angle.
